# Shade avoidance in the context of climate change

**DOI:** 10.1093/plphys/kiad004

**Published:** 2023-01-09

**Authors:** Jorge J Casal, Christian Fankhauser

**Affiliations:** Universidad de Buenos Aires y Consejo Nacional de Investigaciones Científicas y Técnicas, Instituto de Investigaciones Fisiológicas y Ecológicas Vinculadas a la Agricultura, Facultad de Agronomía, 1417 Buenos Aires, Argentina; Fundaciόn Instituto Leloir, Instituto de Investigaciones Bioquímicas de Buenos Aires, Consejo Nacional de Investigaciones Científicas y Técnicas, 1405 Buenos Aires, Argentina; Centre for Integrative Genomics, Faculty of Biology and Medicine, University of Lausanne, CH-1015 Lausanne, Switzerland

## Abstract

When exposed to changes in the light environment caused by neighboring vegetation, shade-avoiding plants modify their growth and/or developmental patterns to access more sunlight. In Arabidopsis (*Arabidopsis thaliana*), neighbor cues reduce the activity of the photosensory receptors phytochrome B (phyB) and cryptochrome 1, releasing photoreceptor repression imposed on PHYTOCHROME INTERACTING FACTORs (PIFs) and leading to transcriptional reprogramming. The phyB-PIF hub is at the core of all shade-avoidance responses, whilst other photosensory receptors and transcription factors contribute in a context-specific manner. CONSTITUTIVELY PHOTOMORPHOGENIC1 is a master regulator of this hub, indirectly stabilizing PIFs and targeting negative regulators of shade avoidance for degradation. Warm temperatures reduce the activity of phyB, which operates as a temperature sensor and further increases the activities of PIF4 and PIF7 by independent temperature sensing mechanisms. The signaling network controlling shade avoidance is not buffered against climate change; rather, it integrates information about shade, temperature, salinity, drought, and likely flooding. We, therefore, predict that climate change will exacerbate shade-induced growth responses in some regions of the planet while limiting the growth potential in others.

## Teaser

The signaling network involved in shade avoidance responses integrates information from environmental variables such as temperature, water availability, and salinity.

##  

### Shade avoidance responses

#### Definition

**Figure 1:**
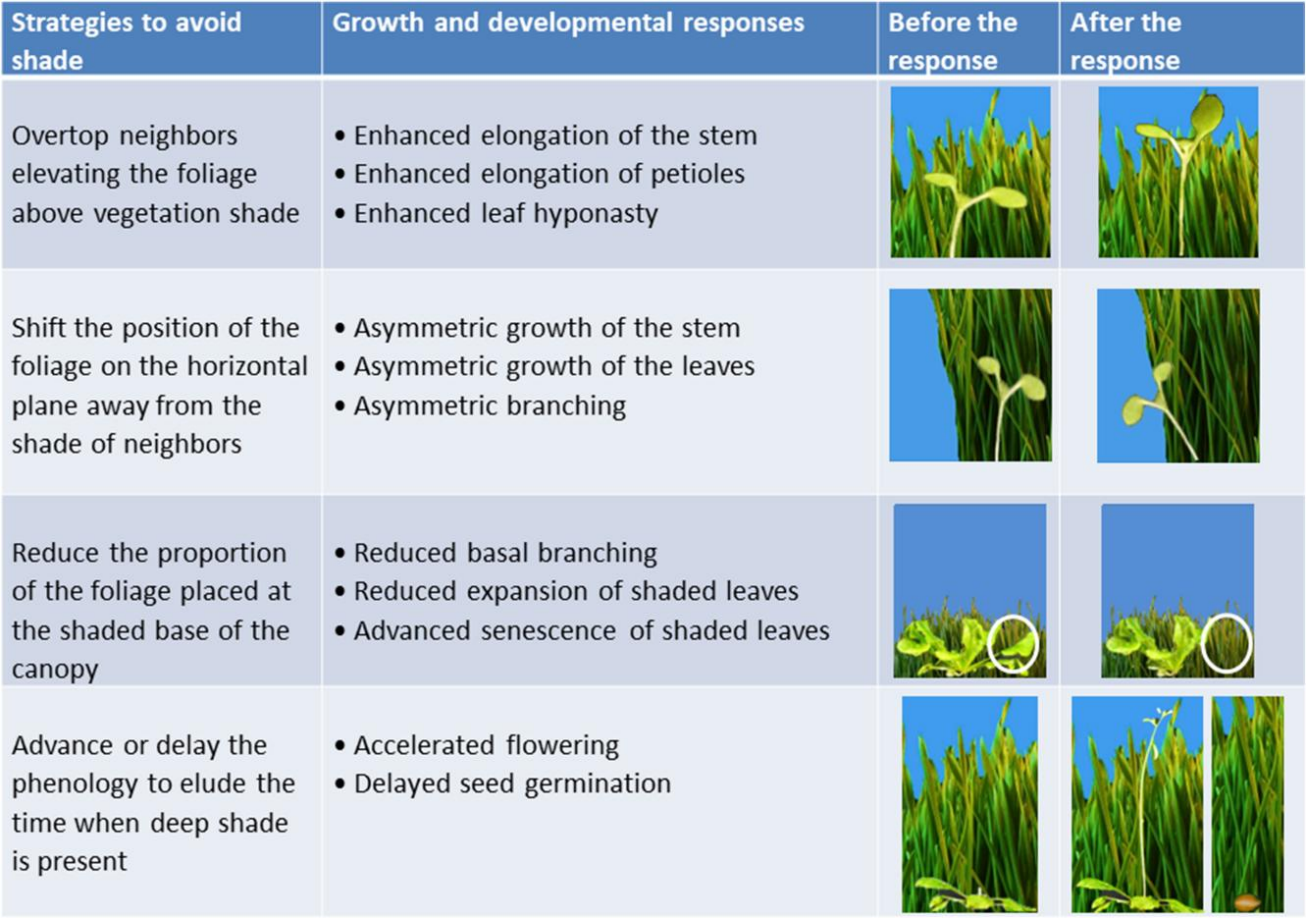
Shade avoidance responses reduce the degree of shade.

Shade avoidance responses are changes in plant growth and/or developmental patterns elicited by modifications of the light environment caused by neighboring vegetation. Their function is to increase access to sunlight and reduce the risk of future shade (Figure 1).

There are four strategies (not mutually exclusive) to achieve these goals: (a) To overtop neighbors, elevating the foliage above vegetation shade; (b) to move the position of the foliage on the horizontal plane away from the shade of neighbors; (c) to reduce the proportion of the foliage placed at the shaded base of the canopy; and (d) to modify the phenology to elude the time when deep shade is present.

The growth and developmental responses inherent to each one of these strategies, respectively, include (a) enhanced elongation of the stem or petioles and the shift of leaves to a more erect position (enhanced leaf hyponasty); (b) asymmetric growth of the stem or the leaves (the phototropic response of light-grown seedlings can be included within this class of shade avoidance responses) and asymmetric branching; (c) the inhibition of branching at the base of the plant, the reduction of expansion of shaded leaves and the advanced senescence of basal leaves; and (d) the accelerated transition to reproduction (flowering) to complete the life cycle before shade becomes too deep and the delayed seed germination until shade disappears.

Shade avoidance responses occur in numerous crop species and are important for agriculture. For instance, neighbor cues increase stem elongation in soybean (*Glycine max*) (Lyu et al., 2021) and sunflower (*Helianthus annuus*) (Libenson et al., 2002), reduce tillering in wheat (*Triticum aestivum)* (Casal, 1988), barley (*Hordeum vulgare*) (Skinner and Simmons, 1993), and sorghum (*Sorghum bicolor*) (Kebrom et al., 2006), and enhance leaf senescence of basal sunflower leaves (Rousseaux et al., 1996) and orient photosynthetic organs on the horizontal plane in maize (*Zea mays*) (Maddonni et al., 2002) and sunflower (López Pereira et al., 2017). Enhanced carbon allocation to the stem accompanies the elongation of this organ in mustard (*Sinapis alba*) and sunflower (Casal et al., 1995; Mazzella et al., 2008). The widely spread idea that crops would benefit from genetically ablating shade-avoidance responses is an oversimplification. Enhanced stem growth can divert resources from harvestable organs and increase the risk of lodging. However, shade avoidance responses also help to optimize canopy architecture in terms of light interception and penetration to lower strata.

Different species may show selected components of the shade-avoidance syndrome and shade avoidance is actually weak in shade-tolerant plants adapted to the understory of tree canopies (Gommers et al., 2013). This review primarily focuses on the responses in shade-avoiding plants. We will present the sensory and signaling mechanisms with an emphasis on Arabidopsis (*Arabidopsis thaliana*) for which the molecular events are best understood. We propose that the output of this network integrates different light, temperature, salinity, drought, and likely flooding cues and is, therefore, affected by climate change.

#### Other responses to neighbor cues

The presence of neighbors modifies diverse features of the physical and chemical environment, including mechanical cues (de Wit et al., 2012; Pantazopoulou et al., 2022), which can be sensed by dedicated receptors. In the context of this article, we refer exclusively to the neighbor cues sensed by photosensory receptors. In addition to the shade avoidance responses described here, changes in the activity of photosensory receptors elicit other plant responses such as photosynthetic acclimation (Cagnola et al., 2012; Morelli et al., 2021), increased water use efficiency (Boccalandro et al., 2009), down-regulation of plant defenses (Pierik and Ballaré, 2021), and altered root growth (Van Gelderen et al., 2018; Rosado et al., 2022). These responses are out of the scope of this review because they do not contribute directly to reduce the intensity of current shade or the risk of future shade (although they might contribute indirectly, by releasing resources for shoot growth and shade avoidance).

### The perception of neighbor cues

#### Progressive intensity of neighbor cues in growing canopies

Compared with isolated plants, the intensity of neighbor cues shows three phases of progressive strength with closer proximity to these neighbors and increased size particularly of their green organs (Casal, 2013). First, far-red light reflected by the green tissues of neighbors reduces the red/far-red ratio while the photosynthetic organs remain fully exposed to light. Second, some plant organs (stem and crown of grass plants) become shaded, but the main photosynthetic organs (leaves) remain fully exposed to sunlight. Third, the photosynthetic organs become shaded. These phases describe the progressive transition from early neighbor detection to the perception of actual shade. Actual shade involves not only low red/far-red ratios but also a low irradiance of UV-B (280–315 nm), UVA (315–400 nm), and photosynthetic radiation (400–700 nm), which includes blue and red wavebands.

#### Phytochrome B (phyB) and cryptochrome 1 (cry1) repress shade-avoidance responses under sunlight

In *A. thaliana*, sunlight activates phyB and cry1 to repress shade avoidance responses. The loss-of-function mutants of these photosensory receptors show shade avoidance responses under full sunlight (Mazzella and Casal, 2001). Quantitatively, phyB makes the strongest contribution (Hernando et al., 2021). There are secondary roles of phyD and phyE, which are more prevalent at certain temperatures and photoperiods (Halliday and Whitelam, 2003), and of cry2 (Mazzella and Casal, 2001).

The biologically inactive form of phyB absorbs red light, which causes its transformation to the active form. The active form absorbs far-red light, which causes its transformation back to the inactive form of phyB (Burgie et al., 2021). In addition, the active form can back-revert spontaneously to the inactive form via thermal reversion. Incoming sunlight contains slightly more red than far-red, and this condition establishes a large proportion of active phyB. Under sunlight, the photochemical reactions of phyB are very fast and the impact of thermal reversion is negligible (Sellaro et al., 2019). The chlorophyll present in green tissues of neighbors absorbs most of the photosynthetic light (400–700 nm) that they intercept. Conversely, green tissues reflect and transmit a large proportion of the far-red light (700–800 nm). Therefore, plants can detect nearby vegetation even before it causes shade (Ballaré et al., 1987) because reflected far-red light reduces the pool of active phyB. Under a plant canopy, the drop in the ratio between red and far-red decreases further. In addition, under shade, the overall irradiance is lower; the photochemical reactions become slower (product of rate constants by irradiance) and, therefore, thermal reversion has a proportionally stronger impact, lowering the proportion of phyB in its active form (Sellaro et al., 2019). Incoming sunlight contains blue light, which activates cry1 (Wang and Lin, 2020). This activity decays under shade due to the absorption of blue light by photosynthetic pigments. The higher proportion of green light under shade is predicted to partially counteract blue-light activation of cry1 in the field (Bouly et al., 2007; Sellaro et al., 2010).

While the drop of phyB and cry1 active pools initiates shade-avoidance responses, other photosensory receptors condition these responses complementing the information about the environment. First, shade avoidance responses on the horizontal plane require sensing the direction of the light input. Neither hypocotyl-phototropism (Goyal et al., 2016) nor leaf-position responses to kin neighbors (Crepy and Casal, 2016) occur in the absence of phototropins, the blue light receptors that provide such cues. Second, daytime activity of phyA and cryptochromes helps to discriminate between a drop in phyB activity caused by neighbors, which elicits shade-avoidance responses, or by the night, which should not trigger a shade-avoidance response (see Casal, 2013 for further discussion).

#### Attenuation of shade avoidance under the canopy

There are two ecologically contrasting conditions that attenuate shade-avoidance responses in plants grown under the foliage of neighbors. One is the penetration of direct sunlight through gaps in the canopy, causing the interruption of shade. The impact of these interruptions depends on their actual duration, the time of the day, and whether they are repeated in successive days (Sellaro et al., 2011; Moriconi et al., 2018). Given the specific kinetics of these interruptions, the contribution exerted through the different photosensory receptors is not the same as described above for the repression of shade avoidance under full sunlight (out of the canopy). For instance, UV-B perceived by UV-B RESISTANT 8 (UVR8) is more effective to inhibit hypocotyl growth in plants grown under low than high red/far-red ratios (Hayes et al., 2014). Therefore, UVR8 effectively reduces the magnitude of shade avoidance when direct light penetrates through gaps in the canopy interrupting periods of low red/far-red ratios (Moriconi et al., 2018).

The other condition that partially attenuates shade avoidance is deep shade. Given its particular mode of action (Rausenberger et al., 2011), phyA differs from other phytochromes because its contribution to the repression of hypocotyl growth is maximal under the low red/far-red ratios (<0.3) found under deep shade and not under full sunlight (Fraser et al., 2021; see Hernando et al., 2021, for a quantitative analysis). Partial repression of shade avoidance under deep shade could be part of a strategy aimed to avoid engaging in a lost competition effort. In favor of this interpretation, the shade-tolerant species *Cardamine hirsuta* (related to *A. thaliana*) shows enhanced expression of the *PHYA* gene and phyA accumulation, which combined with a higher specific intrinsic activity of phyA and enhanced activation of inhibitors of shade avoidance prevents hypocotyl elongation in far-red rich environments (Molina-Contreras et al., 2019; Paulišić et al., 2021).

### The transcriptional network involved in shade-avoidance responses

#### Shade-avoidance responses require PHYTOCHROME INTERACTING FACTORs (PIFs)

**Figure 2:**
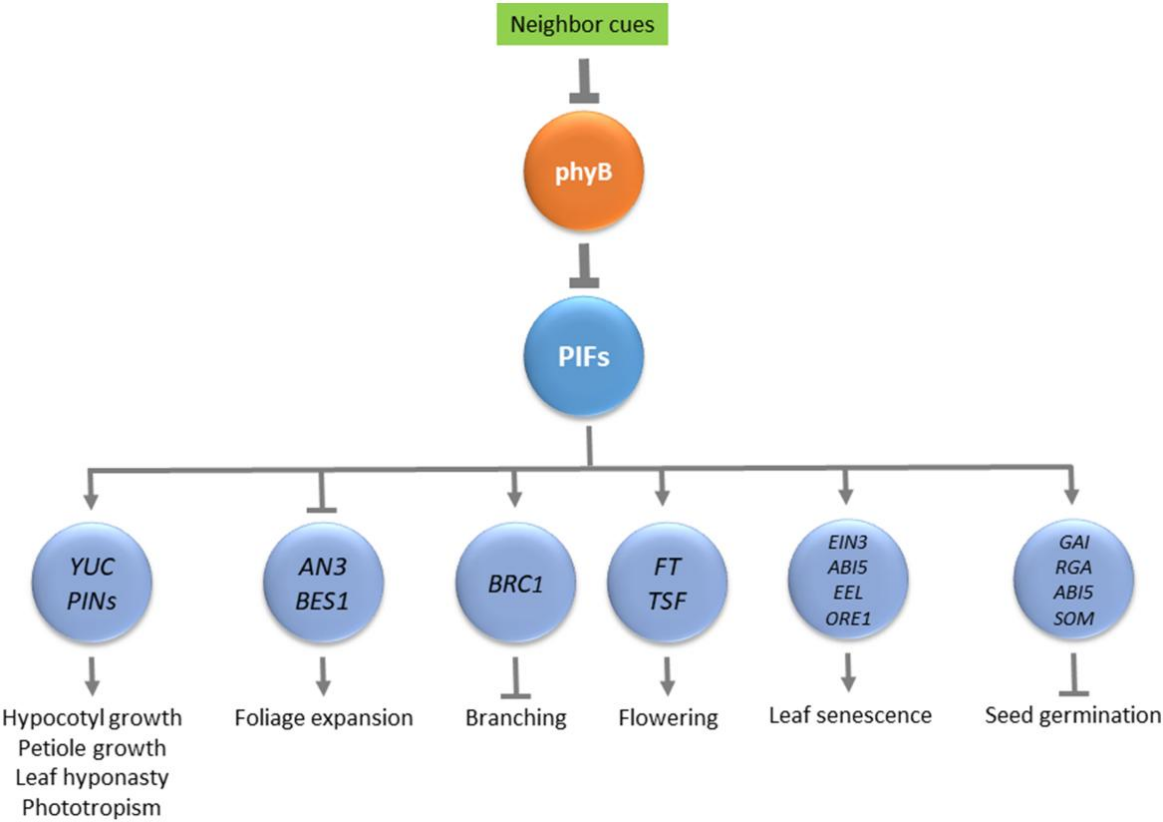
Centrality of the phyB-PIFs module in the control of shade-avoidance responses. phyB and PIFs are involved in all shade-avoidance responses by controlling the expression of a set of response-specific genes. The diagram depicts the only two components demonstrated to operate in all shade avoidance responses (phyB and PIFs) and examples of the genes through which PIFs affect the specific shade avoidance responses supported by genetic evidence. COP1, for instance, is not included because it is very important for hypocotyl growth but not for flowering responses to neighbor cues.

The loss-of-function mutants of *pif4*, *pif5* (Lorrain et al., 2008), *pif7* (Li et al., 2012), and *pif3* (Leivar et al., 2012a; Sellaro et al., 2012) show impaired hypocotyl growth promotion in the presence of cues from neighbors. Petiole growth (Lorrain et al., 2008; de Wit et al., 2015), leaf hyponasty (Michaud et al., 2017; Pantazopoulou et al., 2017), phototropism (Goyal et al., 2016), branching (Holalu et al., 2020), flowering (Galvāo et al., 2019), cotyledon expansion (Costigliolo Rojas et al., 2022), and leaf senescence (Sakuraba et al., 2014) responses to neighbor cues are also impaired in loss-of-function mutants of these transcription factors, while the *pif1* mutant shows poor repression of seed germination (Oh et al., 2004), demonstrating the fundamental role of PIFs in shade avoidance (Figure 2).

#### Neighbor cues release PIFs from the inhibition imposed by phyB and cry1

Under sunlight, phyB and cry1 repress the activity of PIFs and shade alleviates this inhibition. PIFs physically interact with phyB (Pham et al., 2018a). Active phyB assembles into liquidlike droplets by undergoing phase separation and recruits PIFs to these nuclear bodies (Chen et al., 2022). Under white light, nuclear PIF7 colocalizes with phyB in nuclear bodies, and lowering the red/far-red ratio causes the rapid (<30 min) disaggregation of these nuclear bodies toward the nucleoplasm (Willige et al., 2021). By direct physical interaction, phyB facilitates the phosphorylation of PIF3, PIF4, PIF5, and PIF7, which is followed by ubiquitination and degradation in the 26S proteasome; thus, neighbor cues increase the nuclear abundance of all these PIFs (Lorrain et al., 2008; Leivar et al., 2012a; Li et al., 2012; Huang et al., 2018; Pham et al., 2018a; Zhou et al., 2021). UBIQUITIN-SPECIFIC PROTEASE 12 (UBP12) and UBP13 are deubiquitinating enzymes that help stabilize PIF7 and enhance shade-avoidance responses (Zhou et al., 2021). In the case of PIF7, phosphorylation facilitated by phyB favors its interaction with 14-3-3 proteins and cytoplasmic retention (Huang et al., 2018) but as noted above there is nuclear PIF7 even under white light (Willige et al., 2021). In the nucleus, phyB also sequesters PIFs preventing their binding to target promoters and this effect can be dissected genetically from the control of stability. A point mutation in the N-terminal half of phyB impairs the ability of the photosensor to sequestrate PIF3 without affecting its capacity to induce PIF3 degradation whereas the complementary phenotype can be achieved by genetic disruption of the C-terminal half (Qiu et al., 2017; Park et al., 2018). Binding of PIF7 to its target gene promoters is negligible under white light (Willige et al., 2021). Cry1 interacts with PIF4 and PIF5 and reduces PIF4 transcriptional activity, suggesting that the low blue light levels typical of shade release PIF4 from this inhibition (Ma et al., 2016; Pedmale et al., 2016). Although low blue light per se does not have substantial effects on PIF5 levels, the combination of low red/far-red ratios and low blue light (typical of shade) increases PIF5 abundance more than low red/far-red alone (De Wit et al., 2016).

#### PIFs are essential for rapid shade-induced transcriptional reprogramming

The vast majority of genes that respond to low red/far-red ratios depend on the PIFs. (Leivar et al., 2008; Kohnen et al., 2016; Willige et al., 2021; Ince et al., 2022). PIFs bind preferentially to G-boxes (CACGTG) (Hornitschek et al., 2012; Oh et al., 2012; Zhang et al., 2013; Chung et al., 2020; Willige et al., 2021). The histone 2A variant Z (H2A.Z) is enriched specifically in gene bodies and low red/far red ratios cause PIF-dependent removal of H2A.Z at genes that increase their expression (Willige et al., 2021). This effect is rapid and fully reversible 2 h after the seedlings return to high red/far-red ratios. The INOSITOL-REQUIRING MUTANT80 (INO80) chromatin remodeling complex facilitates H2A.Z removal; one of the subunits of the complex interacts with PIFs and mutants of these subunits show reduced promotion of hypocotyl elongation by low red/far-red ratio (Willige et al., 2021). A similar mechanism mediates H2A.Z eviction at PIF4 targets in response to warm temperatures (Xue et al., 2021). Neighbor cues also induce acetylation of the ninth lysine of the histone 3 tail (H3K9) in regulatory and body regions of stimulated genes; a response that requires PIFs (Willige et al., 2021).

#### Other transcription factors

The current information places PIFs at a preeminent position downstream of the photosensory receptors (Figure 2). However, shade-avoidance responses require additional transcription factors, which include, for instance, BRI1-EMS-SUPPRESSOR 1/BRASSINAZOLE-RESISTANT 1 (BES1/BZR1), AUXIN RESPONSE FACTOR 7 (ARF7)/ARF8, TEOSINTE BRANCHED 1, CYCLOIDEA, AND PCF FAMILY 3 (TCP3), TCP5, TCP17, and other bHLH transcription factors. The *bes1* and *bzr1* (Costigliolo Rojas et al., 2022), *arf7 arf8* (Reed et al., 2018), *tcp13 tcp15 tcp5* (Zhou et al., 2018), and *bhlh48 bhlh60* (Yang et al., 2021) mutants show severely impaired hypocotyl growth responses to neighbor cues. The promoters of a large proportion of the genes induced rapidly by neighbor cues in both hypocotyls and cotyledons bear binding sites of PIF5, BZR1, and ARF6 (Kohnen et al., 2016).

Some of these transcription factors can act in parallel to PIFs because they are down-regulated by phyB and/or cry1 and share some of the PIF target genes. For instance, PIFs, ARFs, and BES1/BZR1 form a network of physically interacting transcription factors, with specific and shared gene target promoters and mutually-dependent effects on gene expression at least in the context of the response to warm temperatures (Bai et al., 2012; Gallego-Bartolomé et al., 2012; Oh et al., 2012; Oh et al., 2014). bHLH48 and bHLH60 bind to DNA with poor transcriptional activity but they interact with PIF7 to enhance its DNA-binding activity and bHLH60 shares overlapping genomic targets with PIF7 (Yang et al., 2021). Active phyB interacts with ARF6 and ARF8 and (at least in the case of ARF6) reduces their target DNA-binding capacity (Mao et al., 2020). Also, phyB and cry1 interact with BES1, apparently reducing its target DNA-binding capacity (Wang et al., 2018; Wu et al., 2019). Neighbor cues modulate the stability of both BES1 and BZR1, which increase their nuclear levels in the hypocotyl and decrease them in the cotyledons to mediate the opposite growth responses of these organs (Costigliolo Rojas et al., 2022). bHLH48 and bHLH60 also interact with phyB and neighbor cues increase their protein stability (Yang et al., 2021). The effects of TCP17 involve direct binding to PIF target promoters and simulated shade increases the overall and promoter-bound protein abundance of TCP17 by protecting it from 26S proteasome-dependent degradation (Zhou et al., 2018). In summary, photosensory receptors regulate the stability and/or transcriptional activity of key shade-avoidance transcription factors.

Some of the downstream targets of PIFs are transcription factors themselves (Hornitschek et al., 2012; Leivar et al., 2012b). A conspicuous target of PIFs is *ARABIDOPSIS THALIANA HOMEOBOX PROTEIN 2* (*ATHB2)* (Steindler et al., 1999), which modulates shade responses by mechanisms that have not been fully elucidated. Some of the transcription factors that act in parallel to PIFs also work downstream of PIFs. This is the case of ARFs because PIFs enhance auxin synthesis to promote hypocotyl growth (Tao et al., 2008) and ARFs mediate the transcriptional responses to auxin (Weijers and Wagner, 2016). Also, PIF4 negatively regulates the expression of *BES1* to reduce cotyledon expansion under shade (Costigliolo Rojas et al., 2022).

In addition to the mechanisms of negative regulation of the core transcription factors by phyB and/or cry1 involving stability and/or transcriptional activity, light conditions can also affect *PIF* gene expression levels. For instance, prolonged exposure to low blue light enhances the expression of *PIF4* (Boccaccini et al., 2020). Since TCP17 binds the *PIF4* and *PIF5* promoters to increase the expression of these genes and this pathway accounts for a substantial proportion of its phenotype (Zhou et al., 2018), it is tempting to speculate that TCP17 mediates the cry1 effect on *PIF4* expression. Overexpression of *TCP17* causes constitutive shade avoidance, which depends at least partially on PIFs.

#### Transcriptional regulators form negative feed-forward loops

Several transcriptional regulators reduce the magnitude of shade avoidance responses. They include DELLA proteins (Djakovic-Petrovic et al., 2007), LONG HYPOCOTYL IN FAR-RED (HFR1) (Sessa et al., 2005), PHYTOCHROME RAPIDLY REGULATED1 (PAR1)/PAR2 (Roig-Villanova et al., 2007), PHYTOCHROME INTERACTING FACTOR 3-LIKE 1 (PIL1) (Roig-Villanova et al., 2006), and some INDOLE-3-ACETIC ACID INDUCIBLE (IAA) proteins (Pierik et al., 2009; Pucciariello et al., 2018). DELLA, HFR1, and PAR do not bind DNA but physically interact with PIFs (and some of them also with ARFs and BES1/BZR1) impairing their recognition of target DNA sequences (De Lucas et al., 2008; Hornitschek et al., 2009; Hao et al., 2012). IAAs bind to ARFs, which in turn bind DNA, recruiting transcriptional repressors to the target gene loci (Weijers and Wagner, 2016). Both cry1 and phyB bind to selected IAA proteins increasing their stability and, at least in the case of cry1, IAA stabilization results from reduced interaction with the auxin coreceptor TRANSPORT INHIBITOR RESPONSE 1 (TIR1) (Xu et al., 2018).

Neighbor cues decrease the activity of these negative regulators of shade avoidance by different mechanisms. Interaction with their auxin coreceptor E3 ligases (such as TIR1) targets IAAs to degradation in the proteasome (Weijers and Wagner, 2016) and shade reduces IAA stability (Iglesias et al., 2018). HFR1 and DELLAs are directly targeted for degradation by CONSTITUTIVELY PHOTOMORPHOGENIC1 (COP1, see below). In addition to reduced stability, other proteins sequester negative regulators to reduce their availability to interact with the core transcription factors. The double B-Box (BBX) containing zinc-finger transcription factor BBX24 promotes PIF4 activity by sequestering DELLA proteins (Crocco et al., 2015). The non-DNA-binding basic helix-loop-helix (bHLH) KIDARI (KDR)/PACLOBUTRAZOL RESISTANCE6 (PRE6) increases its expression under low red/far-red ratios to promote shade avoidance by interacting with PAR1 and PAR2, among other partners that reduce hypocotyl growth (Buti et al., 2020).

Noteworthy, several of these transcriptional regulators form negative feedback loops because neighbor cues promote the expression of *HFR1* (Sessa et al., 2005), *PAR1/PAR2* (Roig-Villanova et al., 2006), *PIL1* (Roig-Villanova et al., 2006), and *IAAs* (Leivar et al., 2012b) and increase PIL1 protein stability (Li et al., 2014). Actually, these genes are direct targets of PIFs (Oh et al., 2014; Kohnen et al., 2016; Pedmale et al., 2016). Negative feedback loops typically provide stability. Low red/far-red ratios rapidly and transiently increase the expression of the *HFR1* gene and HFR1 protein accumulation but these effects are weaker when low blue light accompanies these low ratios as observed under shade (De Wit et al., 2016). Thus, HFR1 abundance decreases with more threatening neighbor cues and would, therefore, help to provide a graded physiological output. These regulatory loops could adjust the magnitude of shade avoidance to the characteristics of the neighbor cue such as duration or time of day, other conditions of the environment, organs, etc. For instance, although PIL1 reduces shade avoidance in the long term (Roig-Villanova et al., 2006), it promotes the rapid response to transient drops in red/far-red ratio (Salter et al., 2003).

### COP1 promotes shade avoidance

#### phyB and cry1 inhibit COP1 activity

phyB and cry1 inhibit COP1 activity, hence in shade, when these photoreceptors are less active, COP1 activity increases. COP1 and SUPPRESSOR OF PHYA-105 1 (SPA1) to SPA4 form a complex that acts as an E3 ubiquitin ligase substrate recognition module (Ponnu and Hoecker, 2021). In the light, active phyB and cry1 reduce the activity of COP1 via convergent mechanisms. First, photosensory receptors drive COP1 subcellular localization to the cytoplasm (Subramanian et al., 2004). Second, phyB (Lu et al., 2015; Sheerin et al., 2015) and cry1 (Lian et al., 2011; Liu et al., 2011) repress the activity of the nuclear pool of COP1 by interacting with SPA1, and disrupting the interaction between COP1 and SPA1, which is crucial for the activity of the complex. Furthermore, active cry1 also interacts with COP1 in an SPA1-dependent manner (Holtkotte et al., 2017). The interaction with COP1 occurs via a sequence-divergent Val-Pro motif present in cry1 and COP1 substrates, thereby cry1 acts as a competitive inhibitor of COP1 interaction with targets (Lau et al., 2019). Due to the reduced activity of phyB and cry1 under shade, COP1 increases its nuclear abundance (Pacín et al., 2013) and presumably the nuclear COP1-SPA1 complex increases its intrinsic activity due to the reversal of the aforementioned physical interaction with the photosensory receptors.

#### COP1 reduces the abundance of negative regulators of shade avoidance

COP1 targets some of the negative transcriptional regulators of PIFs, such as HFR1 (Pacín et al., 2016) and DELLA proteins (Blanco-Touriñán et al., 2020), for degradation. The canonical pathway of gibberellins causes 26S proteasome degradation of DELLAs (Sun, 2011) but the increase of gibberellin levels by neighbor cues is too slow (Bou-Torrent et al., 2014) to account for the rapid reduction in DELLAs under shade (Djakovic-Petrovic et al., 2007; Blanco-Touriñán et al., 2020). COP1 has reduced binding affinity to HFR1 of *Cardamine hirsuta*, which is, therefore, more stable and helps reduce shade avoidance in this shade-tolerant species (Paulišić et al., 2021).

#### COP1 controls the stability of transcription factors that induce shade avoidance

COP1 stabilizes positive regulators of shade avoidance responses such as PIF3/PIF4/PIF5 (Pham et al., 2018b) and BES1 (in hypocotyl cells, Costigliolo Rojas et al., 2022) by poorly understood mechanisms. COP1 might target the negative regulators of the stability of these transcription factors (canonical pathway) for degradation and/or reduce the interaction between these transcription factors and their negative regulators (noncanonical pathway, Ling et al., 2017). In addition, COP1 targets BES1 for degradation in the cotyledons generating organ-specific responses to shade (Costigliolo Rojas et al., 2022).

### Organ specificity and intercommunication in shade avoidance

#### Hypocotyl growth

Upon exposure to neighbor cues, the hypocotyl of *A. thaliana* seedlings shows a lag period of about 45 min before elevating its growth rates, followed by a transient drop to intermediate values between 150 and 230 min and the recovery of the high, persistent growth rates (Cole et al., 2011). A drop in fluorescence driven by the DII-VENUS reporter indicates elevated auxin signaling in the hypocotyl 1 h after the beginning of neighbor cues (Kohnen et al., 2016).

Shade-avoidance responses often depend not only on the cues perceived by the responsive organ but also on those perceived by other organs. This dual dependency is likely an adaptation of plants to integrate the heterogeneous light environment and elicit a response adjusted to the perceived threat. For instance, the promotion of hypocotyl growth requires that the cotyledons perceive the neighbor cues (Procko et al., 2014). These cues trigger enhanced activity of PIFs in the cotyledons, which bind and activate the promoter of auxin synthesis genes to elevate the concentration of auxin (Hornitschek et al., 2012; Li et al., 2012) (Figure 2). The auxin synthesis genes *YUCCA 2 (YUC2), YUC5, YUC8*, and *YUC9* are shade induced within 15 min in cotyledons (Kohnen et al., 2016). The quadruple *yuc* mutant lacks shade-induced hypocotyl elongation and cotyledon-specific expression of *YUC3* is sufficient to promote hypocotyl elongation (Kohnen et al., 2016). Auxin travels down to the hypocotyl and is directed by PIN-FORMED (PIN) transporters toward the growth-limiting epidermis to promote the elongation of this organ (Keuskamp et al., 2010; Procko et al., 2014). In the hypocotyl, auxin induces cell-wall acidification to promote cell elongation (Lin et al., 2021).

Auxin synthesis in the cotyledons does not fully account for the hypocotyl growth promotion. First, there are local effects at the hypocotyl. The expression of *YUC8* increases later on in the hypocotyl and might contribute to growth (Kohnen et al., 2016). Furthermore, in epidermal cells of the hypocotyl, low red/far-red ratios reduce the expression of the gene encoding an enzyme involved in auxin conjugation and degradation (Gretchen Hagen 3.17, GH3.17), which reduces hypocotyl growth (Zheng et al., 2016). There are also local effects downstream of auxin levels. Although addition of the auxin analog Picloram fully rescues the *yuc2 yuc5 yuc8 yuc9* quadruple mutant or the *shade avoidance 3* (*sav3*) mutant deficient in auxin synthesis (Tao et al., 2008; Kohnen et al., 2016), multiple *pif* mutants do not reach wild-type levels of hypocotyl growth even when treated with an optimal dose of Picloram, suggesting the occurrence of hypocotyl-specific processes mediated by PIFs (Nozue et al., 2011; Hornitschek et al., 2012; Kohnen et al., 2016). The PIF-dependent promotion of expression of members of the *SMALL AUXIN-UPREGULATED RNA 19* (*SAUR19*) gene subfamily occurs 15–45 min after the beginning of neighbor cues, even in the *pin3 pin4 pin7* and *yuc2 yuc5 yuc8 yuc9* mutants (Kohnen et al., 2016). SAURs promote cell-wall acidification required for growth (Spartz et al., 2014) and the hypocotyl PIFs-SAUR19 pathway may have a role even before auxin from the cotyledons reaches the hypocotyl. Shade-avoidance responses persist under prolonged shade but after the first hours of exposure to neighbor cues, auxin levels return to the prestimulation contents (Bou-Torrent et al., 2014; Pucciariello et al., 2018). The system is then more sensitive to auxin. Prolonged shade elevates the nuclear levels of PIF4 in vascular tissues of the hypocotyl and PIF4 in these tissues per se promotes hypocotyl growth. PIF4 favors the expression of *IAA19* and *IAA29*, which repress the ARF-induced expression of *IAA17*, a strong repressor of hypocotyl growth (Pucciariello et al., 2018). Prolonged shade also increases the abundance of auxin receptors (Pucciariello et al., 2018). The hypocotyl-growth response also requires BES1/BZR1 and these transcription factors increase their nuclear levels specifically in the hypocotyl (Costigliolo Rojas et al., 2022). Since the expression of many genes requires the combined action of PIFs and BES1/BZR1, it is tempting to speculate that these transcription factors have a crucial role in the hypocotyl-specific processes. In summary, hypocotyl pathways involving increased YUC8, SAUR, IAA19/IAA29, and BES1/BZR1 and reduced GH3.17 activities potentially act locally before, during, and after the cotyledon-derived auxin wave.

Second, auxin is not the only signal traveling from the cotyledons as the allocation of additional carbon resources to the hypocotyl accompanies the enhanced elongation of this organ in response to neighbor cues (De Wit et al., 2018). Carbon is primarily transported in the form of sucrose in plants and sufficient sucrose transport capacity is as important as increased auxin production for the rapid induction of hypocotyl elongation by low red/far-red (De Wit et al., 2018). An intriguing open question is the inter-relationship between PIFs, auxin, and sucrose in the control of growth. Higher sucrose levels in seedlings, either due to the inability to produce starch or to exogenous application, enhances hypocotyl elongation in a PIF-dependent manner (Stewart et al., 2011; De Wit et al., 2018). Moreover, more soluble sugars lead to PIF-dependent auxin production and higher PIF levels and/or activity (Sairanen et al., 2012; Lilley et al., 2012; Shor et al., 2017). How these different growth-controlling elements are coordinated requires additional investigations.

Thus, sugars are a signal and the fuel required for hypocotyl growth. In the presence of far-red light reflected by nonshading neighbors the capacity to fix carbon is not jeopardized, but actual canopy shade diminishes CO_2_ uptake, compromising sugar availability. The comparison of gene expression in seedlings exposed either to low red/far-red ratios or to low blue light (which lowers photosynthetic light in addition to cry1 activity) shows that both conditions elicit similar hypocotyl elongation but very different reprogramming of gene expression (Pedmale et al., 2016; Ince et al., 2022). In fact, while the first treatment enhances the expression of genes related to many anabolic processes, the second enhances the expression of genes involved in catabolic processes, including autophagy. Low blue light actually enhances autophagy in cotyledons and hypocotyls and autophagy is required for hypocotyl elongation in shade (Ince et al., 2022). These results suggest that the promotion of hypocotyl growth under shade requires the resources released by autophagy and that the seedlings follow specific metabolic strategies to cover the needs of elongating hypocotyls depending on available carbon resources.

#### Petiole growth and leaf hyponasty

**Figure 3:**
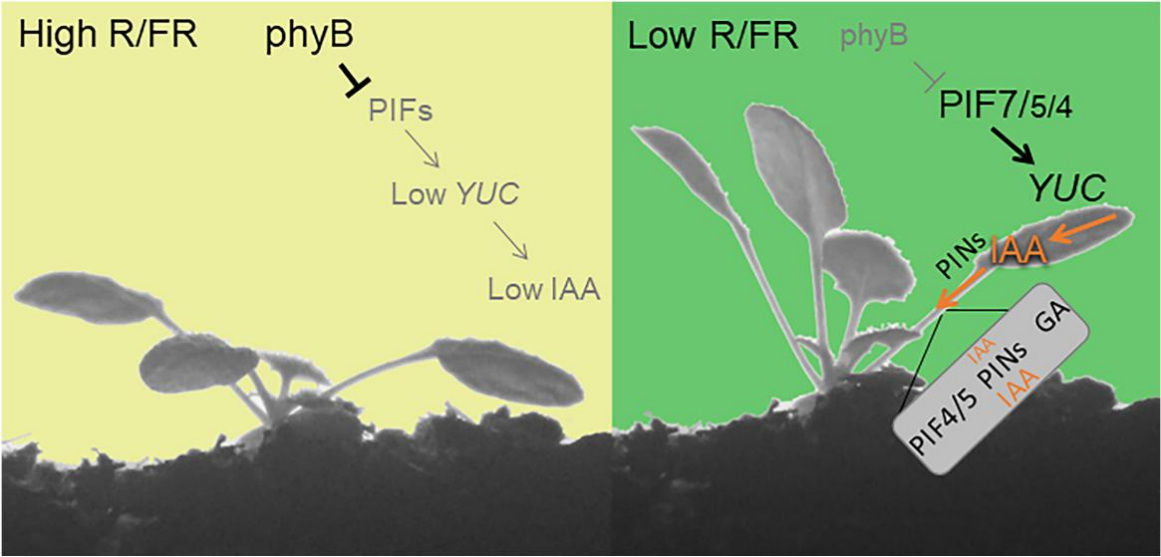
Shade cues can act distally. We illustrate this concept with the case of leaf hyponasty in response to low red/far-red ratios (R/FR) in *A. thaliana*. Under high R/FR active phyB represses leaf hyponasty. Low R/FR releases PIF7, PIF4, and PIF5 from this repression, and these transcription factors induce the expression of auxin (IAA) synthesis and transport genes. The PIN efflux transporters carry IAA from the blade to the petiole and redistribute IAA to the abaxial side of the petiole. There, IAA promotes growth, facilitated by the action of PIF4 and PIF5 and by the synthesis of gibberellin (GA).

The overall mechanisms underlying the elongation and repositioning of the petioles in response to neighbor cues are analogous to those regulating shade-promoted hypocotyl elongation (Nozue et al., 2015; de Wit et al., 2015) (Figure 3). However, in contrast to hypocotyl elongation, petiole elongation requires salicylic acid (Nozue et al., 2018). Resembling the cotyledon-hypocotyl situation, PIF-regulated expression of *YUC* genes in the leaf blade followed by auxin transport to the petiole are key steps leading to petiole growth promotion and upward repositioning (hyponasty) of leaves (Michaud et al., 2017; Pantazopoulou et al., 2017). An important difference between petiole elongation and hyponasty is that for the former response, the site of shade perception and growth promotion can coincide, while for the latter shade, perception must happen in the lamina to trigger upward repositioning of the petiole (Michaud et al., 2017; Pantazopoulou et al., 2017). This is due to the requirement of asymmetric auxin redistribution in the petiole triggering enhanced expansion of the cells on the lower (abaxial) side ultimately leading to hyponasty (Pantazopoulou et al., 2017; Küpers et al., 2023). Intriguingly, petioles also can reposition laterally suggesting that depending on where the shade cue is sensed on the rim of the lamina this leads to upward and lateral repositioning of the leaf away from shade cues (Crepy and Casal, 2015; Michaud et al., 2017). Central to leaf responses is controlled transport of auxin synthesized in the blade, involving several members of the PIN family of auxin efflux carriers (Michaud et al., 2017; Pantazopoulou et al., 2017; Küpers et al., 2023). The plasmodesmata contribute to proper channeling of the growth hormone toward the petiole (Gao et al., 2020). In the petiole, asymmetrical accumulation of auxin elicits gibberellic acid-mediated growth promotion that is stronger on the abaxial side (Küpers et al., 2023). Low red/far-red ratios lead to rapid, PIF-mediated induction of *9-CIS-EPOXICAROTENOID DIOXIGENASE 3 and 5 (NCED3/5)* expression (a rate-limiting enzyme) and higher abscisic acid (ABA) levels (Michaud et al., 2022). Gene expression patterns suggest that the ABA response declines later, which may explain the lower sensitivity to applied ABA in low red/far-red ratios (Michaud et al., 2022). Whether ABA action is restricted to the blade or petiole is unknown but ABA acts in several cell types to allow a full hyponastic response (Michaud et al., 2022). Intriguingly ABA negatively regulates hyponasty in standard (sun mimicking) growth conditions, however, in response to low red/far-red ratios (or higher temperature, which also enhances leaf hyponasty, see below) ABA is needed for a full hyponastic response (van Zanten et al., 2009; Michaud et al., 2022).

#### Phototropism

Positive phototropism in etiolated seedlings is a well-known response allowing plantlets to orient their cotyledons toward the light. Phototropism also occurs in green, photoautotrophic seedlings but it is typically inhibited in sun mimicking conditions (Goyal et al., 2016), except in plants like clover (*Medicago* sp.) or sunflower which perform heliotropism to continuously reposition their leaves according to the solar position (Atamian et al., 2016). Low red/far-red ratios, low blue light, their combination, or actual canopy shade promote phototropism (Goyal et al., 2016; Boccaccini et al., 2020). The mechanisms involve PIF-mediated auxin production through the YUC pathway (Figure 2) and the formation of a steeper auxin gradient across the hypocotyl in shaded seedlings. In sunlight both cry1 and phyB limit the activity of PIFs to prevent a strong phototropic response (Goyal et al., 2016; Boccaccini et al., 2020).

#### Foliage expansion

In the *phyB* mutant of *A. thaliana* plants grown at 21°C, the rosette leaves show diminished expansion due to a combination of reduced cell proliferation at early stages of leaf development, and reduced cell expansion at later stages (Romanowski et al., 2021). Similarly, lowering phyB activity by a pulse of far-red light at the end of each daily photoperiod reduces cell division only if the treatments started early, while late treatments more effectively reduced cell size (Romanowski et al., 2021). Far-red light releases PIF7 from the inhibition imposed by phyB and then PIF7 directly represses the expression of *ANGUSTIFOLIA3* (*AN3*) and hence of the AN3 targets *GROWTH REGULATING FACTOR1 (GRF1)*, *GRF3*, and *GRF5* involved in cell proliferation (Hussain et al., 2022) (Figure 2). An additional pathway controls leaf cell proliferation apparently by reducing the levels of cytokinin. In fact, low red/far-red ratios increase the expression of the *CYTOKININ OXIDASE 6 (CKX6)* gene involved in the breakdown of cytokinin, a response proposed to be mediated by enhanced auxin levels in leaf primordia (Carabelli et al., 2007).

Neighbor cues reduce cotyledon expansion in young seedlings (Josse et al., 2011; Li et al., 2012; Procko et al., 2014), offering a model to study the mechanisms of foliar cell expansion without the involvement of cell division, which is negligible in this context. Simulated shade reduces the nuclear abundance of BES1 and BZR1 in the cotyledons, two transcription factors that promote the expansion of this organ (Costigliolo Rojas et al., 2022). The latter is the result of two convergent pathways initiated by the enhanced levels of PIF4 and COP1 in response to shade lowering phyB activity in the cotyledons. PIF4 reduces the expression of *BES1* (Figure 2) whereas COP1 physically interacts with BES1 inducing its degradation in the 26S proteasome pathway (Costigliolo Rojas et al., 2022).

#### Branching

Low red/far-red ratios or the *phyB* mutation reduce branching in *A. thaliana* (Finlayson et al., 2010; González-Grandío et al., 2013). The *pif4 pif5* mutant background partially alleviates the effects of the *phyB* mutation or of low red/far-red ratios on branching (Holalu et al., 2020). PIF4/PIF5 inhibits bud outgrowth by a combination of local and systemic mechanisms. Locally, PIF4/PIF5 promotes the expression of *BRANCHED 1 (BRC1)* (Holalu et al., 2020) a known repressor of branching (González-Grandío et al., 2013). In turn, BCR1 binds and activates the promoters of the *HOMEOBOX PROTEIN 21 (HB21), HB40*, and *HB53* genes and these transcription factors, together with BRC1, increase the expression of *NCED3* (Gonzalez-Grandio et al., 2017) (Figure 2). NCED3 is involved in ABA synthesis and increases the levels of this hormone in the bud to repress its outgrowth (Reddy et al., 2013; Gonzalez-Grandio et al., 2017; Holalu et al., 2020). There is also a systemic effect of low red/far-red ratios or the *phyB* mutation, revealed by the correlative inhibition of bud outgrowth, which is stronger for buds closer to the shoot apex (Finlayson et al., 2010). This systemic effect involves auxin in the polar auxin transport stream (Krishna Reddy and Finlayson, 2014) and PIF4/PIF5 increase the sensitivity to auxin under conditions where phyB activity is low (Holalu et al., 2020).

#### Flowering

Light cues indicative of dense plant populations also alter developmental transitions such as entry into the reproductive phase. In Arabidopsis, such light cues accelerate the transition to flowering, whereas the opposite occurs in alfalfa (*Medicago sativa*) (Lorenzo et al., 2019). The Arabidopsis response depends on expression of the floral inducer *FLOWERING LOCUS T* (*FT*) and its paralog *TWEEN SISTERS OF FT* (*TSF*) (Kim et al., 2008b; Schwartz et al., 2017). As *FT* expression is controlled by several endogenous and exogenous cues including ambient temperature and vernalization, accessions with different vernalization requirements respond differently to changes in the red/far-red ratio (Adams et al., 2009). In rapid cyclers (such as Col) low red/far-red ratios accelerate flowering in inductive photoperiods. Like growth adaptations, this developmental response depends on PIF7 with contributions of PIF4 and PIF5 (Galvāo et al., 2019; Zhang et al., 2019) (Figure 2). Available evidence supports a model where PIFs cooperate with the photoperiodically controlled CONSTANS (CO) transcription factor to directly regulate the expression of *FT* and *TSF* (Galvāo et al., 2019; Zhang et al., 2019). COP1 affects CO stability (Jang et al., 2008) but the enhanced *FT* expression and accelerated flowering in response to neighbor cues do not require COP1 or SPAs (Rolauffs et al., 2012).

#### Leaf senescence

The analysis of leaf senescence in light-grown seedlings transferred to darkness indicates that the process is repressed by phyB and promoted by PIF4 and PIF5, which enhance the expression of the senescence gene *ORESARA1* (*ORE1*) by direct binding to its promoter (Sakuraba et al., 2014). In addition, PIF4 and PIF5 enhance *ORE1* expression indirectly by binding to and enhancing the expression of *ABA INSENSITIVE 5 (ABI5)*, *ENHANCED EM LEVEL (EEL)* and *ETHYLENE INSENSITIVE 3 (EIN3)* followed by direct activation of ORE1 by these transcription factors (Sakuraba et al., 2014). These molecular mechanisms may operate in the context of shade-induced leaf senescence (Figure 2), but their relevance remains untested.

#### Seed germination

Although PIF1, also known as PIL5, plays a very minor role in shade-avoidance responses involving seedlings and adult plants, it is a strong repressor of seed germination (Oh et al., 2004). In the seeds, the direct targets of PIF1 include the DELLA genes *GIBBERELLIC ACID INSENSITIVE* (*GAI*) and *REPRESSOR OF ga1-3* (*RGA*) (Oh et al., 2007), *ABI5* (Oh et al., 2009) and the gene encoding the nucleus-localized CCCH-type zinc-finger protein SOMNUS (*SOM*) (Kim et al., 2008a), all of which contribute to repress seed germination by favoring the impact of ABA compared with gibberellin signaling. The disruption of the canopy cover by large herbivores, wind impact, etc., exposes the seeds to unfiltered sunlight, activating phyB to promote seed germination (Botto et al., 1996). Active phyB reduces the stability of PIF1 tipping the balance in favor of gibberellin signaling (Oh et al., 2006). In addition, active phyB interacts directly with the transcription factors ETHYLENE RESPONSE FACTOR 55 (ERF55) and ERF58, reducing their binding to activate the *PIF1* and *SOM* promoters (Li et al., 2022).

### Shade avoidance and climate change

#### Global warming and deeper shade

In many areas of the planet, plants are experiencing a temperature increase that is so fast that it jeopardizes their ability to adapt. Moreover, global temperatures will continue to rise to an extent that will depend on the adoption of strong climate actions (Battisti and Naylor, 2009). In parallel, a current strategy to increase the yield of agricultural crops is to elevate the number of plants per unit soil area (Cagnola et al., 2021) a practice that increases mutual shading among plants. There is strong resemblance between the growth responses of young Arabidopsis seedlings exposed to neighbor cues or to warm, nonstressful (<30°C) temperatures (Quint et al., 2016; Casal and Balasubramanian, 2019). Both show long hypocotyls and reduced cotyledon area compared with seedlings grown at ambient temperatures (≈20°C) in the absence of neighbor cues. Like shade, warm temperatures also increase petiole growth and hyponasty and accelerate flowering in Arabidopsis.

#### Mechanistic convergence of the growth responses to shade and warmth

**Figure 4:**
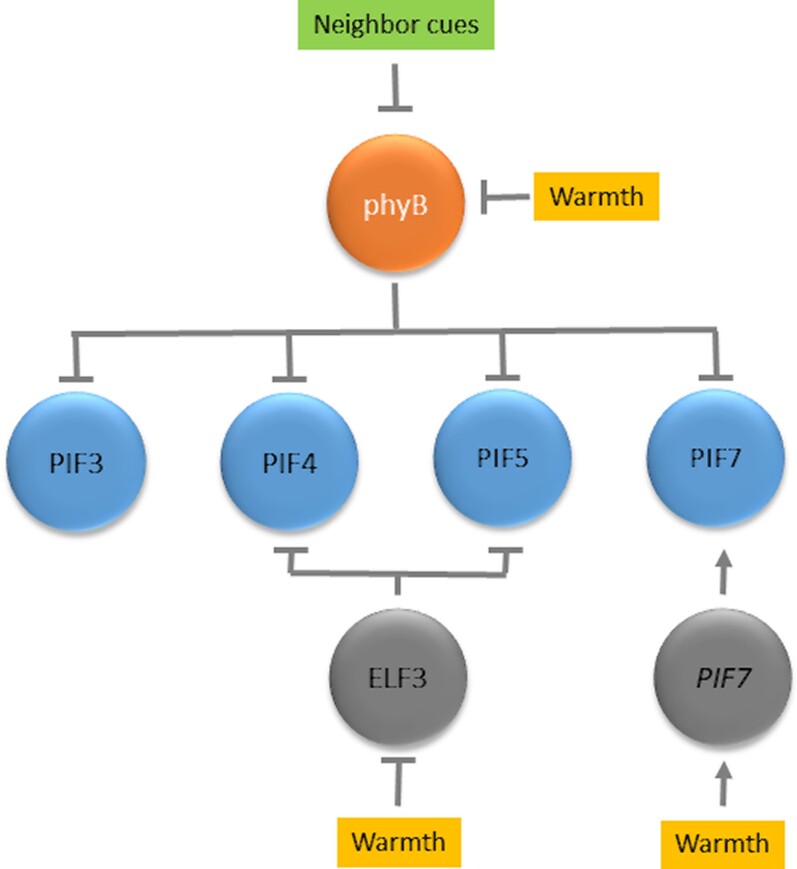
The phyB-PIFs module integrates shade and warmth information. Warm temperatures cause thermal reversion of the active to the inactive form of phyB, cause liquid–liquid phase separation of ELF3 reducing the binding of the EVENING COMPLEX that it integrates to the *PIF4* and *PIF5* promoters to reduce their activities and modifies the structure of the *PIF7* mRNA hairpin increasing its translation.

The signaling networks of these responses share key components (Figure 4). For instance, the activity of phyB not only decreases in response to neighbor cues but also in response to warm temperatures because thermal reversion from its active to its inactive form increases with temperature within the physiological range (Jung et al., 2016; Legris et al., 2016). Therefore, the pathways repressed by phyB are partially released from this inhibition by elevated temperatures. In addition, temperature affects components of the shade avoidance network by other mechanisms. Warmth increases the expression of the *PIF4* gene (Koini et al., 2009). The evening complex represses the expression of *PIF4* (Nusinow et al., 2011) and elevated temperatures reduce the binding of the complex to the *PIF4* promoter (Silva et al., 2020). EARLY FLOWERING 3, one of the components of this complex, is a temperature sensor that undergoes liquid–liquid phase separation under warm temperatures, affecting the activity of the complex (Jung et al., 2020) (Figure 4). Warmth increases PIF7 nuclear abundance (Chung et al., 2020; Fiorucci et al., 2020) and/or the proportion of dephosphorylated PIF7 (Burko et al., 2022). Warm temperatures modify the structure of the RNA hairpin present at the 5′-untranslated region of the *PIF7* transcript (a third temperature sensor), increasing its rate of translation and hence PIF7 protein abundance (Chung et al., 2020) (Figure 4). Like neighbor cues, warm temperatures increase the synthesis of auxin in the cotyledons, which travels to the hypocotyl (Franklin et al., 2011; Sun et al., 2012; Bellstaedt et al., 2019). During the early promotion of hypocotyl growth, the quantitative impact of different *pif* mutations on the response to shade and on the response to warmth is very similar (Romero-Montepaone et al., 2021).

Side by side comparison of the transcriptome indicates that shade and warmth converge to promote the expression of many of the same growth-related genes, reflecting their shared hypocotyl growth phenotype (Romero-Montepaone et al., 2021). However, they also diverge with specific effects of temperature on the expression of genes involved in thermotolerance, some of which decrease their expression in response to shade. Another interesting divergence is observed for HFR1 because shade promotes *HFR1* expression while reducing HFR1 protein stability (see above), whereas warmth has little effect on expression and increases protein stability (Romero-Montepaone et al., 2021). HFR1 minimizes adverse effects of elevated temperature on plant growth (Foreman et al., 2011).

#### Synergism between shade and warmth

When combined, shade and warmth have synergistic effects on hypocotyl growth (Romero-Montepaone et al., 2020; Burko et al., 2022). PIF4 and PIF7 are important for this synergism. In the case of PIF4, its nuclear abundance in hypocotyl cells increases synergistically and the quantitative relationship between hypocotyl growth rate and PIF4 levels is similar when PIF4 is increased by shade, warmth, or their combination (Romero-Montepaone et al., 2021). The synergism in PIF4 levels could result from the effect of warm temperature on *PIF4* expression in combination with a stronger effect of shade on phyB levels and hence on PIF4 stability. The mechanisms of PIF7 in the synergic response remain obscure (Burko et al., 2022). Modeling predicts that as a result of this synergism, shade avoidance will become more intense with continued global warming (Romero-Montepaone et al., 2020). Other shade-avoidance responses could likely share the synergic pattern reported for hypocotyl growth, but this pattern should not be considered a rule. In specific contexts, strong activation of the shared network by shade could leave little room for the action of warm temperature, or vice-versa, and the combined effects could be less than additive.

#### Functional convergence of growth responses to shade and warmth

Shade increases the magnitude of hypocotyl growth and leaf hyponastic responses to warm temperatures (Vasseur et al., 2011; Romero-Montepaone et al., 2021). The combination of low irradiances (typical of actual shade) and warm temperatures can deteriorate the carbon budget (including the contents of sucrose and starch) more than any of the two factors in isolation (Vasseur et al., 2011). In fact, elevated temperatures can reduce photosynthesis and increase respiration exacerbating the problems in carbon balance imposed by shade. As a result of this, achieving the compensation point requires more light, i.e. a stronger shade-avoidance response, when combined with warm temperatures (Romero-Montepaone et al., 2021). Therefore, the synergism between shade and warmth may serve the purpose of intercepting more light.

#### Drought, salinity, and flooding effects on shade avoidance

Climate change not only affects crop yields via direct consequences of warming but also by increasing the incidence of drought (Naumann et al., 2021), flooding (Chagas et al., 2022), and salinity stresses (Corwin, 2021). In summary, the future scenario may involve changes in the aboveground environment such as more shade and elevated temperatures and changes in soil variables such as reduced water or oxygen availability and increased salinity, among other problems.

Even low levels of salinity decrease the magnitude of shade-avoidance responses (Hayes et al., 2019). This effect requires ABA signaling and involves the reduction of the promotion by neighbor cues of the expression of *BRASSINOSTEROID SIGNALING KINASE 5* (*BSK5*), necessary for BES1 activity (Hayes et al., 2019). Salinity increases oxidative stress and exposure to sunlight would further increase this risk, suggesting that a brake to the shade-avoidance response may be part of a conservative strategy.

The combination of drought and high population densities is seriously detrimental for most Arabidopsis accessions grown in field trials (Exposito-Alonso et al., 2019). Shade avoidance can exacerbate the negative consequences of drought by exposing the foliage to sunlight and consequently increasing its transpiration rate. Water restriction caused by the addition of polyethylene glycol to the substrate to simulate drought conditions reduced the expression of *PIF4*, *PIF5*, and *PIF3* and consequently, the promotion of hypocotyl growth by shade (Semmoloni et al., 2022). This is a specific response because water restriction did not affect cotyledon expansion and its response to shade. CIRCADIAN CLOCK ASSOCIATED 1 (CCA1) and LATE ELONGATED HYPOCOTYL (LHY) associate with the *PIF4* promoter to enhance its expression during the morning (Sun et al., 2019). This mechanism is involved in drought-regulated *PIF4* expression because this response required *CCA1* and *LHY* and water restriction also reduced the activities of these gene promoters. The abundance of the PIF4 protein reflected the changes in *PIF4* gene expression (Semmoloni et al., 2022). Intriguingly, ABA did not mediate this response.

As in the case of warm temperature and shade, there is phenotypic and signaling convergence between plant responses to submergence and shade. Submergence triggers ethylene signaling, which enhances the abundance of PIF3 to promote hypocotyl elongation (Wang et al., 2020). The transcriptome changes induced by ethylene and shade show strong overlap (Das et al., 2016). Therefore, submergence could affect shade avoidance responses.

#### Does shade avoidance modify the impact of climate change on plants?

The shifts in plant architecture caused by shade avoidance responses modify the light profile and hence the temperature profile within the canopy. In turn, this is predicted to affect the rates of transpiration and net carbon dioxide exchange of the foliage present at different canopy depths. Thus, in addition to the effects of climate change on shade avoidance summarized in previous paragraphs, shade avoidance could in principle modify the impact of climate change on plants. This idea remains unexplored but a recent study shows that light reaching the understory of grasslands has system-level consequences (Eskelinen et al., 2022), giving credit to this possibility.

### Conclusions

The phyB-PIF regulon controls the expression of a variety of specific targets to modulate plant growth and development according to population density. Both phyB and PIFs participate in each one of the shade-avoidance responses and other photosensory receptors and transcription factors complement the functionality of this core module. COP1 appears as the major positive regulator of the activity of PIFs reducing the abundance of its negative regulators and/or reinforcing their action via parallel pathways.

Plants are able to detect their neighbors with precision, well before mutual shading takes place (Ballaré et al., 1987; Smith et al., 1990). A priori it was reasonable to assume that this highly sensitive sensory system was buffered against changes in other factors of the environment (e.g. ambient temperature) to maintain the same relationship between stimulus and response. In contrast, the phyB-PIF regulon integrates information from diverse external cues that act at multiple levels of the pathway from the phyB sensor to events occurring downstream of the PIFs. This multilevel integration of information presumably contributes to the flexibility of the system. We are only beginning to uncover this sophisticated signal integration network that the current scenario of climate change urges us to understand (see Outstanding Questions).

**Advances**

- The characterization of different growth and developmental responses in Arabidopsis has elucidated the architecture of the shade avoidance network, where the phyB-PIFs module constitutes the core and other photosensory receptors (cry1, UVR8, and phyA), transcriptional regulators (ARFs and BES1/BZR1), and post-transcriptional regulators (COP1, DELLAs, HFR1, etc.) fulfill crucial organ- and/or environment-specific functions.
- The importance of shade avoidance of liquid–liquid phase separation, chromatin remodeling, and carbon allocation is beginning to emerge.
- The activity of key components of the shade avoidance network responds not only to neighbor cues but also to temperature, water availability, salinity, and/or oxygen availability, acting as cellular integrators of above- and belowground information.

**Outstanding questions**

- To what extent is the signaling network involved in shade avoidance responses in Arabidopsis conserved in other species?
- What is the specific function of the negative regulators of shade-avoidance responses that increase their expression in response to neighbor cues?
- Which genes provide organ specificity to core shade-avoidance proteins such as PIFs or COP1?
- What are the patterns and mechanisms of integration of the auxin and sugar signals moving from the cotyledons to the growing hypocotyl? Is a similar convergence observed for other interorgan communication processes such as control of bud outgrowth?
- Is there deep signaling integration between soil resources-sensing mechanisms affected by climate change (water, oxygen, and nutrients) and the perception of neighbor cues by aerial organs?
- Can shade avoidance responses modify the impact of climate change on plants?
